# Left ventricular remodeling following myocardial infarction revealed with a quantitative diffusion MRI tractography framework

**DOI:** 10.1186/1532-429X-14-S1-P35

**Published:** 2012-02-01

**Authors:** Choukri Mekkaoui, Shuning Huang, Guangping Dai, Timothy G Reese, Aravinda Thiagalingam, Pal Maurovich-Horvat, Jeremy Ruskin, Udo Hoffmann, Marcel P Jackowski, David E Sosnovik

**Affiliations:** 1 Harvard Medical School, Athinoula A. Martinos Center for Biomedical Imaging, Charlestown, MA, USA; 2 Radiology, Massachusetts General Hospital, Harvard Medical School, Boston, MA, USA; 3 Cardiology Division, Massachusetts General Hospital, Harvard Medical School, Boston, MA, USA; 4 Computer Science, University of São Paulo, Institute of Mathematics and Statistics, São Paulo, Brazil

## Summary

A cardiac-tailored framework for 3D Diffusion Tensor MRI tractography is developed and used to characterize myofiber architecture in normal and remodeled myocardium. We show that myofibers in the subepicardium of the remote infarct zone become less oblique (more circumferential) as the heart dilates and remodels. This fiber realignment may play an important role in the loss of contractile function in the remote zone over time.

## Background

Large infarctions cause the left ventricle (LV) to dilate and remodel, ultimately leading to a global decrease in LV function. While the impact of infarction on fiber architecture in the infarct zone is well understood, the impact of remodeling on fiber architecture in the remote zone is not. Diffusion Tensor Imaging (DTI) has contributed greatly to the study of cardiac structure and organization, but the majority of these studies employed 2D datasets [1,2]. Here we aim to characterize fiber architecture in the remote zone of remodeled hearts using a novel 3D cardiac-tailored diffusion MRI tractography framework that allows fibers in the entire heart to be evaluated as continuous entities.

## Methods

Excised human (n=5) and sheep (n=5) hearts were studied. Large anteroseptal infarctions were produced in the sheep 3 months prior to imaging. DTI of the hearts was performed on a 3T scanner (Trio, Siemens) using 6 gradient-encoding directions, b-value=2000s/mm^2^; voxel-size=2x2x2mm^3^; TR/TE=8430/96ms. Fiber tracking was performed with a fourth-order Runge-Kutta approach. Myofibers were classified by their median helix angle (HA) and limited in length to half the LV circumference. Histograms of fiber HA were used to compare myofiber architecture in normal and remodeled myocardium.

## Results

**Figure 1:**
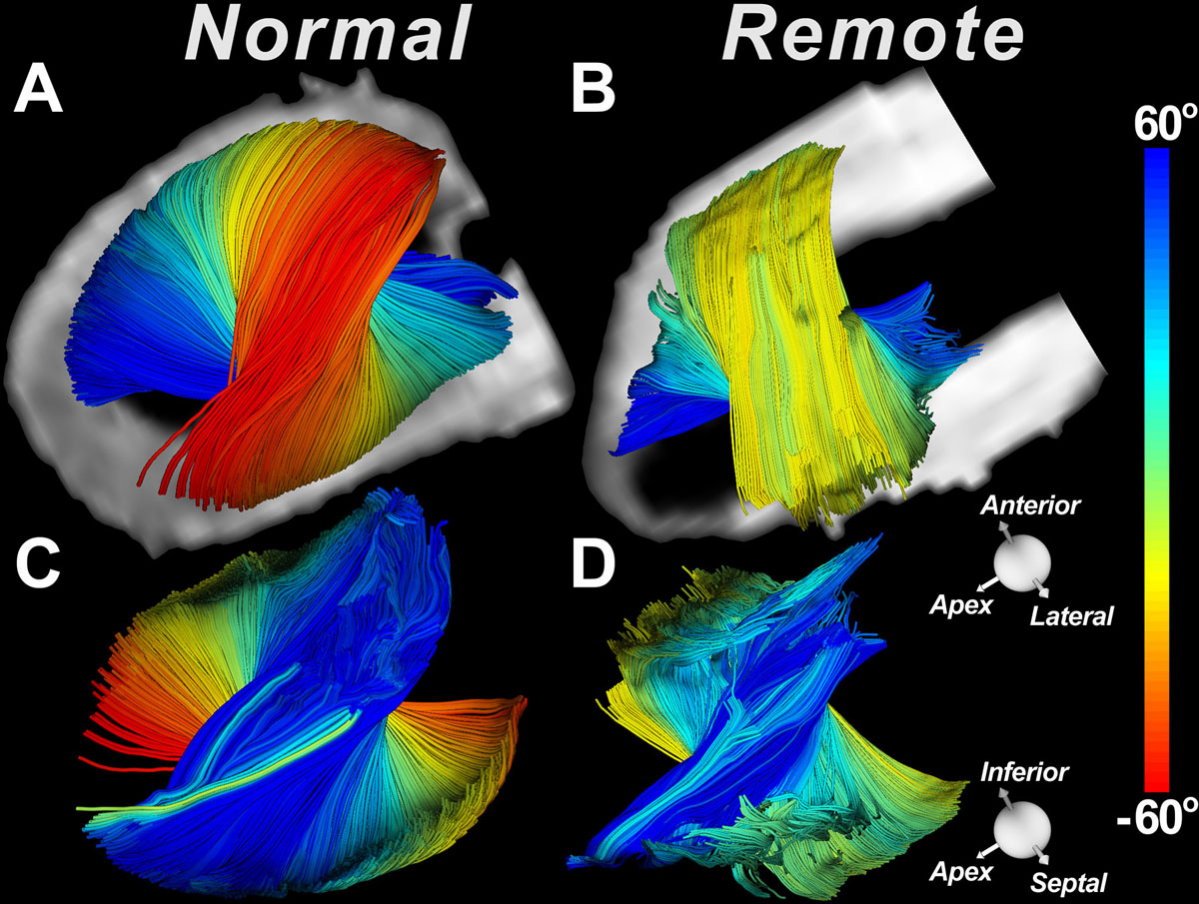
Quantitative tractography of the myocardium: myofibers in the lateral wall of a human heart (**A**) and the remote zone (lateral wall) of a sheep heart with a large anteroseptal infarct (**B**) are shown. The tracts are color-coded by their median helix angle and are being viewed from their epicardial surface. (**C-D**) The tracts have been vertically rotated by 180 degrees and are being viewed from their endocardial surface. Fibers in the subepicardium of the remote zone have undergone a positive (right-handed) shift and become more circumferential.

**Figure 2:**
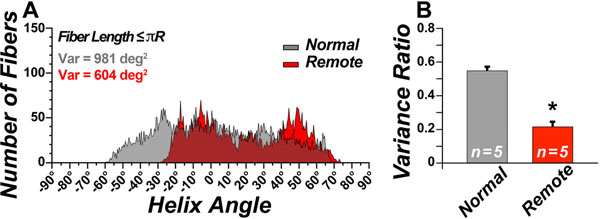
(**A**) Histogram of median myofiber HA in the lateral wall of normal hearts (gray) and hearts with large anteroseptal infarcts (red). (**B**) The ratio of variance between fibers with negative HA and positive HA. The reduction of the variance ratio in the remote zone indicates a loss of negative (left-handed) myofibers.

DTI tractograms in the lateral wall of a normal human heart (Figure 1A,1C) and sheep heart with a large anteropseptal infarct (Figure 1B,1D) are shown. The tracts are being viewed from their epicardial (Figure 1A,1B) and endocardial surfaces (Figure 1C,1D), respectively. Profound thinning of the infarct and dilation/remodeling of the LV is seen in the sheep heart. LV remodeling did not significantly affect fiber organization in the subendocardium. However, in the sheep with large infarcts, myofibers in the subepicardium of the remote zone consistently underwent a rightward (more circumferential) rotation. This was confirmed by a reduction in HA variance (Figure 2A) and in the HA variance ratio: ratio of variance between fibers with negative HA and positive (Figure 2B).

## Conclusions

The developed DTI tractographic framework allows myofibers be to classified as continuous entities and shows that fibers in the subepicardium of the remote zone lose their oblique orientation. This microstructural change likely contributes significantly to the loss of function seen in the remote zone over time.

## Funding

R01 HL093038 (Sosnovik), NCRR P41RR14075 (Martinos Center) and MGH-ECOR (Mekkaoui).
